# Prevention of Tendon Adhesions by ERK2 Small Interfering RNAs

**DOI:** 10.3390/ijms14024361

**Published:** 2013-02-21

**Authors:** Hongjiang Ruan, Shen Liu, Fengfeng Li, Xujun Li, Cunyi Fan

**Affiliations:** Department of Orthopaedics, Shanghai Sixth People’s Hospital, Shanghai Jiaotong University, School of Medicine, 600 Yishan Road, Shanghai 200233, China; E-Mails: ruanhongjiang@126.com (H.R.); liushensjtu@sjtu.edu.cn (S.L.); fengmale@yahoo.com.cn (F.L.); lixujun1983@126.com (X.L.)

**Keywords:** tendon repair, adhesion, siRNA, extracellular signal-regulated kinase (ERK) 2, fibroblast proliferation, lentivirus

## Abstract

Tendon adhesions are one of the most concerning complications after surgical repair of flexor tendon injury. Extracellular signal-regulated kinase (ERK) 2 plays crucial roles in fibroblast proliferation and collagen expression which contributes to the formation of tendon adhesions after flexor tendon surgery. Using a chicken model, we have examined the effects of a small interfering RNA (siRNA) targeting ERK2 delivered by a lentiviral system on tendon adhesion formation with an adhesion scoring system, histological assessment, and biomechanical evaluation. It was found that ERK2 siRNA effectively suppressed the increase of fibroblasts and the formation of tendon adhesions (*p* < 0.05 compared with the control group). Moreover, no statistically significant reduction in breaking force was detected between the ERK2 siRNA group and the control group. These results show that the lentiviral-mediated siRNA system is effective in preventing tendon adhesion formation but not to tendon healing, and may be used for tendon repair after confirmation and improvement by future detailed studies.

## 1. Introduction

Tendon injuries are the second most common hand injuries in trauma and orthopedic patients [1]. In most cases, surgical repair or transplantation is required [2]. However, as a result of an inflammatory response at the surgical site, and the loss of physical separation, local tendon adhesions, as one of the most concerning complications in tendon repair, may form between the tendons and the surrounding sheath [3]. Usually, tendon adhesions bind the flexor tendons to each other or to their sheath, which restricts normal tendon gliding and consequently leads to poor functional recovery. A number of biological or synthetic materials, such as amniotic membrane, Seprafilm and polytetrafluoroethylene membrane, have been evaluated as mechanical barriers to prevent tendon adhesions [4–6]. In addition, the inhibitory effects of pharmacologic agents, such as 5-fluorouracil and hyaluronic acid [3,7,8], on adhesion formation have been investigated as well. Although these studies have obtained some improvements in materials used in surgical repair, due to their complexity and restriction, no reliable therapy has been established. Therefore, it is necessary to develop novel practical approaches to clinically prevent adhesion formation.

RNA interference (RNAi) is an evolutionarily conserved process in which cells employ small interfering RNA (siRNA) duplexes to destroy target messenger mRNAs, so as to silence the activity of corresponding genes [9,10]. The process exists in a variety of organisms and is used to regulate many diverse cellular processes. As a novel and revolutionary approach, RNAi has been rapidly and extensively used in basic biological research and the research and development of drugs and therapies, and has demonstrated great value, owing to its high specificity and potency [11–13]. Nevertheless, whether RNAi can be applied in tendon repair to suppress, or block, adhesion formation remains unclear.

Previous studied have shown that fibroblast proliferation and collagen expression play important roles in the formation of tendon adhesions [14,15]. In our previous work, we demonstrated that extracellular signal-regulated kinase (ERK) 2 is involved in the regulation of collagen expression and fibroblast proliferation induced by transforming growth factor (TGF)-b1 and fibroblast growth factor (FGF)-2 [16]. Furthermore, the effect of the intra-articular administration of a siRNA, targeting ERK2 on joint adhesion formation, has been investigated in a rat model through lentiviral-mediated RNA interference, and the results indicated that local delivery of this siRNA effectively diminishes joint adhesion formation [17]. All these lines of evidence suggest that lentiviral-mediated ERK2 siRNA may be applied in tendon repair, to provide hints for prevention of tendon adhesion formation. Therefore, in this study, the effect of ERK2 siRNA on adhesion formation was investigated in a chicken model of tendon repair.

## 2. Results and Discussion

The lentiviral vector was successfully constructed (data not shown) and the high-titer lentiviral supernatants (for ERK2 siRNA and MS (a mis-sense) siRNA) were obtained for follow-up experiments in a chicken model.

### 2.1. Lentiviral-Mediated ERK2 siRNA Expression

**Figure 1:**
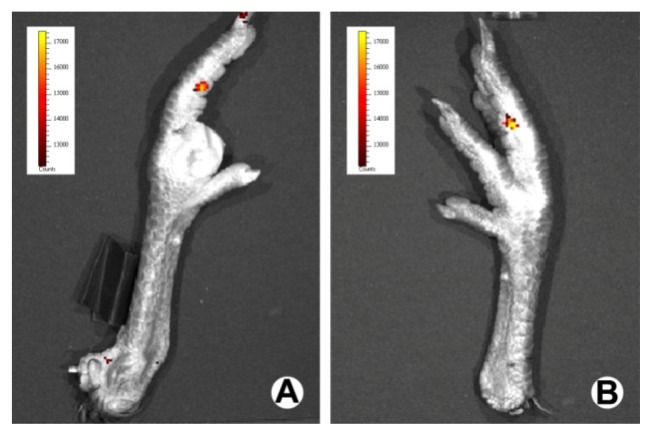
Bioluminescent imaging of a representative chicken foot of the ERK2 siRNA group at 14 (**A**) and 28 (**B**) days after surgical repair.

Fourteen days after surgical repair, bioluminescent imaging demonstrated that the administration of the lentiviral-mediated siRNA system resulted in a localized expression of luciferase around the repaired flexor digitorum profundus (FDP), which also represented the distribution of ERK2 siRNA (Figure 1A). In addition, similar bioluminescent images were obtained in the luciferase fluorescence measurement carried out after 28 days (Figure 1B).

### 2.2. Effective Silencing of ERK2 and pERK2 by Lentiviral-Mediated RNA Interference

**Figure 2:**
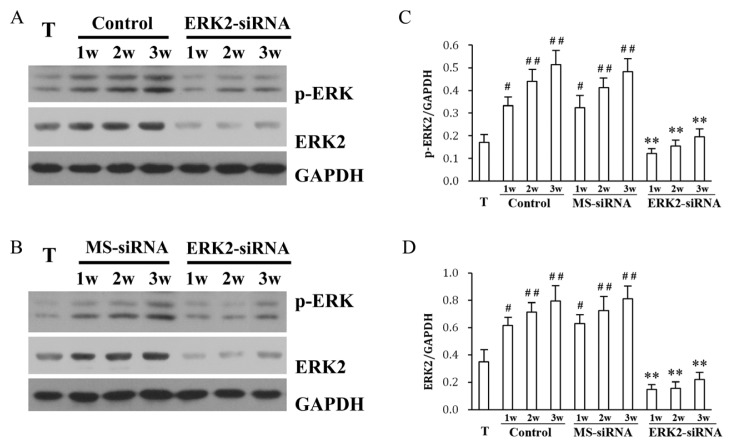
Effect of ERK2 siRNA on ERK2 and p-ERK levels. ERK2 and phosphorylated ERK1/2 in normal tendon tissues and adhesion tissues from the control group, ERK2 siRNA group (**A**) and MS siRNA group (**B**) were detected by Western blotting using specific antibodies at the indicated weeks after surgical repair. Quantification results of the p-ERK2 (**C**) and ERK2 (**D**) bands are shown. Results are means ± SD from three independent experiments. # means *p* < 0.05, ## means *p* < 0.01 *vs.* the T group, ** means *p* < 0.01 *vs.* the Control group.

In the control group, the background level of ERK2 and p-ERK2 in normal tendon tissue was relatively low (Figure 2). Western blot analyses revealed that ERK2 siRNA but not MS-siRNA treatment evidently inhibited ERK2 expression in adhesion tissue of the tendon adhesion model (Figure 2A,B). The phosphorylation of ERK levels were increased significantly in adhesion tissue of the tendon adhesion model in a time-dependent manner (Figure 2). ERK2 siRNA significantly reversed increased p-ERK2 levels in the adhesion tissues, similar to its effect on ERK2 expression (Figure 2A,C,D). On the contrary, the MS siRNA showed no obvious effect on the ERK or p-ERK2 levels (Figure 2B,C,D).

### 2.3. Effective Inhibition of Peritendinous Adhesions by Lentiviral-Mediated ERK2 siRNA

**Figure 3:**
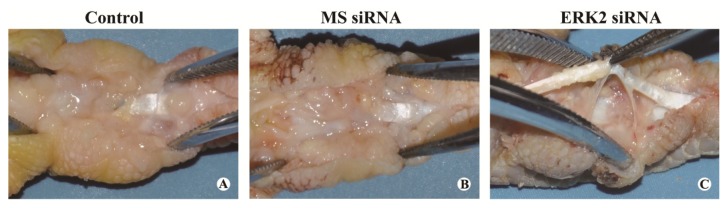
Effect of ERK2 siRNA on adhesion formation. Representative photographs of tendon adhesions in the control group (**A**), MS siRNA group (**B**) and ERK2 siRNA group (**C**) are shown.

**Figure 4:**
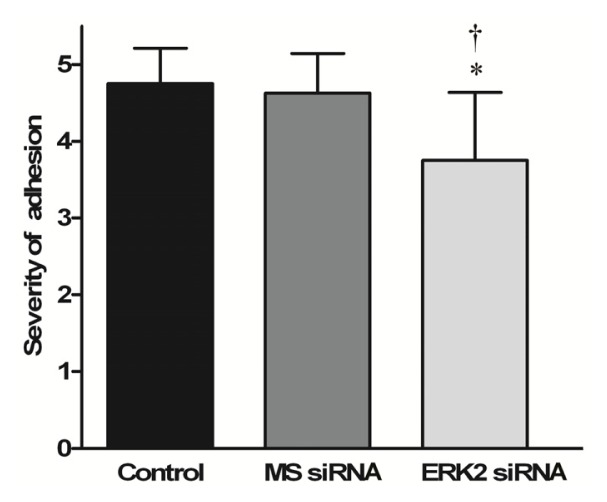
Effect of ERK2 siRNA on adhesion scores. Scores of tendon adhesions in the control group, MS siRNA group and ERK2 siRNA group are presented as mean ± SD (*n* = 8). (* *p* < 0.05 *vs.* Control, ^†^*p* < 0.05 *vs.* MS siRNA).

At 28 days after the surgery, the peritendinous adhesions at the repaired tendons were assessed by visual examination based on a scoring system. Obvious fibrous adhesions were observed between the repaired chicken tendons and the peritendinous tissues in the control and MS siRNA groups (Figure 3A,B). For the tendons treated with lentiviral-mediated ERK2 siRNA, fewer and weak adhesions were observed at the repaired sites (Figure 3C), which could also be separated easily. The scoring results suggest that ERK2 siRNA significantly inhibited the adhesion formation, compared with the control and MS siRNA groups (Figure 4).

**Figure 5:**
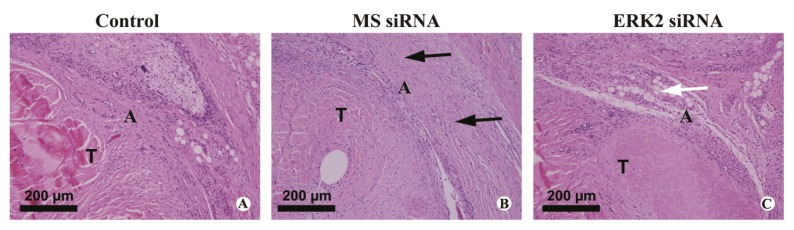
Histological observation of repaired tendon sections at 28 days after tendon repair surgery. Representative microscopic photos of HE stained sections of tendon adhesions in the control group (**A**), MS siRNA group (**B**) and ERK2 siRNA group (**C**) are shown. A: Adhesiontissue; T: Tendon. White arrow indicates scattered weak fiber formation surrounding the tendon (T) while black arrows indicate the dense adhesion tissue.

**Figure 6:**
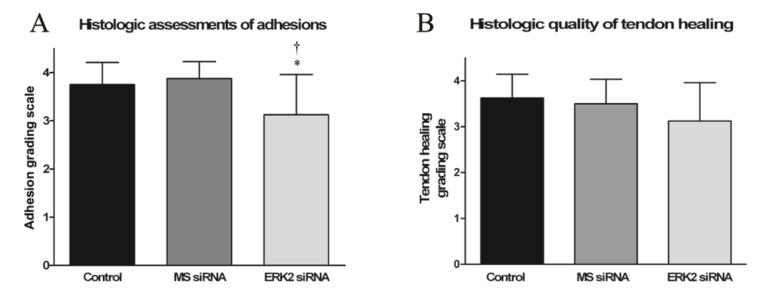

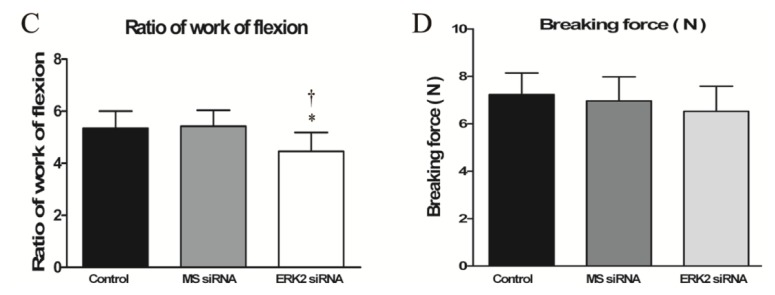
Histological evaluation and biomechanical analysis of repaired tendon sections at 28 days after tendon repair surgery. Scores of histological assessments of adhesions (**A**), histological quality of tendon healing (**B**), ratio of work of flexion (**C**) and the breaking forces of tendons (**D**) are presented as mean ± SD (*n* = 8). Asterisk indicates * means *p* < 0.05 *vs.* Control and ^†^ means *p* < 0.05 *vs.* MS siRNA

Histological results of the hematoxylin and eosin (HE) stained sections of the repaired tendons are shown in Figure 5. Thick fibrous adhesion tissues developed at the repair sites in the control and MS siRNA groups (Figure 5A and B). There was no clear peritendinous adhesion but a little scattered weak fiber formation around the repaired tendons in the ERK2 siRNA group (Figure 5C). The higher concentration of nuclei at the repair sites in the control and MS siRNA groups indicated evident proliferation of fibroblasts, but lower cell density was observed in the ERK2 siRNA treated chickens. Histological assessment of adhesion formation in all treatment groups are shown in Figure 6A. In comparison with the control group and MS siRNA group, the adhesions in the ERK2 siRNA group were significantly lower (*p* < 0.05) (Figure 6A). However, ERK2 siRNA treatment demonstrated no significant effect on the average scores of histological quality of tendon healing (Figure 6B).

### 2.4. Effect of ERK2 siRNA on Biomechanical Properties of Repaired Tendons

Compared to the control and MS siRNA group, a significant decrease in the ratio of work of flexion was observed in the ERK2 siRNA group, indicating reduced peritendinous adhesions (Figure 6C). By contrast, there was no significant difference between the breaking forces in the ERK2 siRNA or MS siRNA group and the control group (Figure 6D).

Similar to the joint adhesions and abdominal adhesions, the formation of tendon adhesion involves fibroblast migration and proliferation and collagen expression that are mediated by TGF-b1, IGF-1 and other growth factors [18,19]. The adhesion formation is closely related to the healing of the tendon. It is well established that both intrinsic healing and extrinsic healing, simultaneously, play roles in the healing process after tendon injury. The extrinsic healing is characterized by an evident inflammatory response followed by specialized fibroblast recruitment and proliferation. To inhibit the problematic tendon adhesion formation, and improve the healing quality of the tendon repaired, it is important to restrain the extrinsic healing and promote the intrinsic healing [20]. The migration of fibroblasts of the paratenon plays an important role in adhesion formation. Increased expression of TGF b1 induces excessive fibroblast proliferation and reduced functionality. TGF b1 also mediates collagen expression in fibroblasts through the SMAD and ERK pathways. The ERK pathway has been proven to contribute to SMAD-mediated signaling and Ras-dependent cell signaling in some cells [21,22]. In addition, recent researches have suggested that ERK2, but not ERK1, plays a dominant role in cell proliferation [23–25]. All aforementioned evidence indicates the ERK2 may act as a crucial mediator in fibroblast proliferation and collagen production, and the consequent formation of tendon adhesions. Moreover, our previous studies have confirmed that the siRNA targeting ERK2 mediated by lentivirus could effectively reduce the proliferation and collagen expression of rat joint adhesion tissue fibroblasts and decrease joint adhesion formation effectively [16,17]. Due to the similar characteristics of the joint adhesion and tendon adhesion, in the present study, the effect of this siRNA was investigated in the flexor tendon repair model.

Taking its advantages into consideration, such as extensively diverse target cells, high infection efficiency, a capacity to hold long sequences, stable expression due to gene integration into the host cells, and the weak immunogenicity and toxic response [26–31], the lentiviral-mediated siRNA delivery system was used in this study to evaluate the effects of ERK2 siRNA on the formation of flexor tendon adhesions. *In vivo* bioluminescent results show that the lentiviral system could efficiently express the siRNA targeting ERK2 in the peritendinous tissues of the checks, indicating a successful local delivery of siRNA, which avoids side effects caused by overall down-regulation of the target gene. Furthermore, owing to lentiviral integration, the ERK2 siRNA delivery system can steadily affect peritendinous fibroblasts for a long time, so that repeated administrations are not required. Taken together, it is indicated that the lentiviral system may be a safe and durable delivery system for gene regulation.

To assess the efficiency of this siRNA delivery system, a chicken model of flexor tendon repair was used. In the control group, evident peritendinous adhesions were found, suggesting that the model was appropriately established. The histological results showed the lentiviral system effectively delivered the ERK2 siRNA to the repair sites and remarkably suppressed adhesion formation. The significantly attenuated formation of tendon adhesions by the administration of ERK2 siRNA was confirmed by biomechanical evaluation as well, in comparison with the control group and MS siRNA group. However, no statistically significant difference in breaking forces was noted between the three groups.

In summary, this is the first study to evaluate the effects of ERK2 siRNA in flexor tendon adhesions and the results demonstrate that ERK2 siRNA effectively inhibited the formation of tendon adhesions. Detailed studies should be carried out to provide more relevant evidence and information for the technique in tendon repair.

## 3. Experimental Section

### 3.1. Lentiviral Vector Construction, Virus Production and Infection

The pshRNA-H1-Luc lentivector purchased from System Biosciences was used in this study, in order to express target siRNA and luciferase originated from the copepod together. The siRNA which had been used to down-regulate ERK2 in rat in our previous work effectively [16], was used to inhibit ERK2 in chicken, since these ERK2 genes bear the identical sequence 5′-GTGATGAGCCTGTAGCTGA-3′. The MS negative control siRNA (5′-CGTTAGTTAGCAGTGAGCG-3′) was also included. The synthesized oligonucleotide templates were annealed and inserted into the linear lentivector. The constructed vectors were transfected into 293TN producer cells with pPACK Packaging Plasmid Mix (System Biosciences, Mountain View, CA, USA) using Lipofectamine™ 2000 (Invitrogen, Carlsbad, CA, USA) in accordance with the manufacturer’s instructions. 48 hours later, the viral supernatants were collected, and cleared by centrifugation and 0.45 μm PVDF membrane filter. Gradient dilution was used to determined viral titers.

### 3.2. Animal Model

All procedures and handling of the animals were carried out in accordance with the policies of Shanghai Jiao Tong University, School of Medicine and the National Institutes of Health. Leghorn chickens (1.5–2 kg each) were used for this study. They were anesthetized by intramuscular injection of ketamine hydrochloride (50 mg/kg). Then, sterile skin preparation and an elastic tourniquet were applied. A lateral skin incision was created on the proximal phalanx of the third toe. After incising the flexor tendon sheath, the FDP was isolated, transversely incised and then repaired using a modified Kessler tendon repair with 6–0 prolene suture (Ethicon Ltd., Edinburgh, UK). The animals were randomly assigned to three groups. In groups I and II, MS siRNA or ERK2 siRNA was injected around the repair site of the FDP, while no treatment was performed before wound closure in the control group. After skin closure, the extremity was immobilized in a weight-bearing splint.

### 3.3. *In Vivo* Bioluminescence Assay

Bioluminescence assays comprise a high-sensitivity and non-invasive technique for monitoring specific cellular and genetic activities in a living organism. At 14 and 28 days after surgical manipulation, the luciferase expression and distribution in the individual chickens, in the ERK2 siRNA group, were measured using a Xenogen IVIS 50 Bioluminescence System (R&D Systems).

### 3.4. Western Blotting

The adhesion tissues from the three groups, and the normal tendon tissues were dissected and homogenized in RIPA lysis buffer. Protein concentrations were determined using BCA assay. Equal amounts of proteins were separated by SDS-PAGE, and transferred onto PVDF membranes (Millipore, MA, USA). The membranes were blocked in TBST containing 5% nonfat milk at room temperature for two hours and incubated with primary antibodies against ERK2 (1:400; Santa Cruz, Santa Cruz, CA, USA), p-ERK (1:500; Santa Cruz, Boston, MA, USA) or GAPDH (1:2000; Santa Cruz, Santa Cruz, MA, USA) at 4 °C overnight. Membranes were then incubated with corresponding HRP-conjugated secondary antibodies against mouse (1:4000; Cell Signaling Technology, Boston, MA, USA) or rabbit IgG (1:3000; Cell Signaling Technology, Boston, MA, USA) at room temperature for one hour. The bands were visualized with an enhanced chemiluminescence reagent (Amersham Biosciences, Sunnyvale, NJ, USA). ERK2 and pERK2 levels were quantified and normalized to GAPDH bands by densitometry.

### 3.5. Macroscopic Evaluation

Before sacrificing the animals, the repair site was visually examined for signs of inflammation or ulceration. The severity of peritendinous adhesion was evaluated by a scoring system [32]. To evaluate the severity of peritendinous adhesions, an adhesion scoring system was used to grade a particular area into grades of 1–5, based on the surgical findings: grade 1, no adhesion; grade 2, adhesion area can be separated by blunt dissection alone; grade 3, adhesion area less than or equal to 50% which required sharp dissection for separation; grade 4, 51%–97.5% adhesion area which required sharp dissection for separation; and grade 5, more than 97.5% of the adhesion area requiring sharp dissection for separation.

### 3.6. Histological Evaluation of Adhesion Tissues

The third toes were fixed in 4% paraformaldehyde for one day and then decalcified in 10% EDTA for one month at room temperature. Samples were dehydrated through increasing concentrations of ethanol and then paraffin embedded. Sections were cut in 4-μm sagittal slices and stained with hematoxylin-eosin(HE). Histologic assessments of adhesions and tendon healing were performed [33]. Adhesions were quantified into four grades as follows: grade 4, severe (>66% of the tendon surface); grade 3, moderate (33%–66% of the tendon surface); grade 2, mild (<33% of the tendon surface); or grade 1, no adhesions. Tendon healing was quantified into four grades as follows: grade 4, poor (failed healing or massive overgrowth of granulation tissue); grade 3, fair (irregularly arranged and partly broken intratendinous collagen bundles); grade 2, good (intratendinous collagen bundles exhibited good repair, but the epitenon was interrupted by adhesions); or grade 1, excellent (good tendon continuity and smooth epitenon surface). These histological sections were evaluated under light microscopy (LEICA DM 4000 B) by two independent investigators blinded to the treatment.

### 3.7. Biomechanical Evaluation

To evaluate peritendinous adhesions and tendon healing, the work of flexion and the breaking force were both measured using a rheometer (Instron 5548, Instron, Norwood, MA, USA). To evaluate the work of flexion, the proximal end of the FDP tendon was fixed to a force gauge and the proximal phalanx of the toe was attached to a home-made device with the proximal interdigital joint fixed by stainless steel rods. The load (Newtons) and the displacement (mm) were measured when the FDP tendon was pulled at 20 mm/min until the angle of the distal interdigital joint was 40°. The work of flexion was then calculated by curve integration. To avoid individual variation, both the repaired and the intact tendons of both sides in each animal were evaluated and the ratio of repaired work of flexion *vs.* intact work of flexion was used as a parameter to determine the difference among different groups. To evaluate breaking force, the repaired chicken FDP tendons were harvested. The proximal and distal ends of the tendon were fixed to the force gauge of the rheometer. The tendon ends were pulled apart at a speed of 20 mm/min until rupture of the tendon occurred, and breaking force was recorded by the rheometer.

### 3.8. Statistical Analysis

Results are expressed as mean ± standard deviation (SD). Statistical software SPSS 10.0 (Chicago, IL, USA) was used to analyze the data by one-way analysis of variance; *p* < 0.05 was considered significant.

## 4. Conclusions

The present study demonstrated that in a chicken flexor tendon repair model, the lentiviral-mediated siRNA targeting ERK2 substantially inhibited peritendinous adhesion formation. This finding may provide a potential novel therapeutic intervention which can be used to eliminate or reduce the formation of postoperative tendon adhesions in the treatment of hand tendon injury.

From the anti-adhesion results of lentiviral-mediated siRNA targeting ERK2, we can learn that the mitogen-activated protein kinase pathway may be involved in the peritendinous adhesion formation. However, further studies of this mechanism and the effect of inflammatory component are needed.
